# Miscellaneous

**Published:** 1876-06

**Authors:** 


### Miscellaneous.

### TREATMENT OF STAMMERING.

One of our dailies contains a letter, which appears trustworthy, written by a gentleman who stammered from childhood almost up to manhood, and who gives a very simple remedy for the misfortune:—"Go into a room where you will be quiet and alone, get some book that will interest you, and sit down and read two hours aloud, to yourself, keeping your teeth together. Do the same thing every two or three days, or once a week if very tiresome, always taking care to read slowly and distinctly, moving the lips but not the teeth

Then, when conversing with others, try to speak as slowly and distinctly as possible, and make up your mind that you will not stammer. Well, I tried this remedy, not having much faith in it, I must confess, but willing to do almost anything to cure myself of such an annoying difficulty. I read for two hours aloud, with my teeth together. The first result was to make my tongue and jaws ache, that is, while I was reading, and the next to make me feel as if something had loosened my talking apparatus, for I could speak with less difficulty immediately. The change was so great that every one who knew me remarked it. I repeated the remedy every five or six days for a month, and then at longer intervals, until cured."

---

Good English authorities estimate the preventable deaths arising from impure air and water in that country at 15,000 annually.

---

### TO DISGUISE TANNIN.

Dr. U. B. K., of Pa.—How can the astrigent taste of *tannin* be overcome, without affecting its medical virtues, when dissolved in water acidulated with acetic or muriatic acid?"

Professor J. M. Maisch kindly furnishes us the following reply to the above:—

"In answer to the query as to how the taste of tannin can be disguised, I have to say that if a complete masking of the astringent taste is intended, I no not believe that can be attained; but if the astringency is to be modified so as to become palatable, I do not think the task is very difficult. Cinnamon, cloves, sassafras bark and other aromatic drugs contain considerable amounts of tannin, yet their astringent taste is not unpleasant—it being modified by the aromatic principles. A large amount of tannin is found in wild cherry bark, yet the cold infusion is not very unpleasant, owing to the bitter almond taste developed, but the taste of the syrup is more agreeable on account of the sugar contained therein.

"The agents best adapted for modifying the astringency of tannin, therefore, appear to be aromatics and sugar, used separately or combined. This plan has been adopted in several fluid extracts and syrups of the United States Pharmacopœia, and is well illustrated by the aromatic syrup of galls, used to some extent in this city, and which contains in 8 fluid ounces the tannin and aromatic principles of 4 drachms of nutgalls and 2 drachms each of cinnamon and mace. This syrup contains over 15 grains of tannin in the fluid ounce, but is readily taken by infants."

### LISTER'S TREATMENT OF WOUNDS BY THE ANTISEPTIC METHOD.

The Following lecture, from the *Lancet*, March 25th, 1876, we give somewhat more at length than usual, on account of the great interest of the subject. It is by Thomas Smith, F. R. C. S., Surgeon to St. Bartholomew's Hospital:

The theory, or one may now say the facts on which Mr. Lister's antiseptic treatment rests, are as follows:—

1st. That in the dust of the atmosphere and on matter with which it is in contact, there are the germs of minute organisms which, under favorable circumstances, induce putrefaction in fluids and solids capable of that change, in the same manner as the yeast-plant occasions the alcoholic fermentation in a saccharine solution.

2d. That putrefaction is not occasioned by the chemical action of oxygen or any other gas, but by the fermentative agency of these organisms.

3d. That the vitality or potency of the germs can be destroyed by heat, and by various chemical substances, which we call, in surgery, "antiseptics."

Now I am not going to ask you to believe these statements on my authority, but I will shortly refer to the results of experiments performed by Pasteur, Lister, Sanderson, Tyndall and others, which justify the above conclusions.

It is scarcely necessary to state that organic fluids, like milk, urine and blood, infusion of meat, etc., if kept in contact with the air at ordinary temperatures, will, ere long, decompose and putrefy, and will give evidence of putrefaction by turbidity (if the fluid be originally clear), by the evolution of offensive gasses, and by the development within them of bacteria.

Further, it has been shown by Pasteur and other observers, that it is by no means essential to the success of such experiments that the organic liquids should be boiled, but that when circumstances admit of their being withdrawn uncontaminated from their natural receptacles, such as the urinary bladder, the blood vessels, the udder of the cow, or the shell of a fresh laid egg, they will remain free from organisms and from putrefaction when kept in pure vessels and protected from dust.

It has also been discovered that impure air will purify itself by mere subsidence of its dust. Pasteur long ago proved that putrescible fluids could be kept free from putrefaction in air taken from cellars free from draughts, when the solid particles of the atmosphere had had time to deposit themselves by subsidence; and Prof. Tyndall has recently subjected air purified by being kept at rest to very searching tests, to ascertain if it will excite putrefaction in putrescible solutions. He has found that solutions of meat, cheese, turnip, etc., first subjected to a high temperature, can be kept free from putrefaction for an indefinite time, exposed to the air-closed boxes that have been kept at rest a day or two, to allow the dust to subside, precautions being taken to prevent the said dust rising again by coating the inside of the box with glycerine. The same experimenter has demonstrated the fact that the air which has been thus rendered incapable of exciting putrefaction—*i. e.*, aseptic—is also optically pure: that is, that there are no particles or motes to be detected in it when illuminated by a beam of electric light in a darkened room.

On the other hand, Mr. Lister has found that when any portion of apparatus used in investigations on this subject can not conveniently be purified by heat, the object may be attained by washing the glass or other material with a strong, watery solution of carbolic acid, and drying it with a carbolized rag, and in the course of a long series of experiments he has invariably found this antiseptic agent as efficacious as the flame of a spirit lamp in preventing the growth of organisms and the occurrence of putrefaction.

Mr. Lister's object in the treatment of wounds and abscesses is to exclude from them these germs or organisms that float in the atmosphere and are the causes of putrefaction, and the means he employs for effecting this purpose he recommends, not as the best that can be used, but as the best that he has been able up to the present time, to devise; and although Mr. Lister considers the truth of his theory incontrovertible, yet he does not claim to have brought his practice to perfection.

Mr. Lister claims for his plan that when it can be carried out with due care and proper observance of details, he can, as a rule, secure that an open wound should heal after the manner of a subcutaneous injury—that is, without inflammation or constitutional fever, and for the most part without suppuration; while, if suppuration occurs, he secures that it shall not be putrefactive—that is, accompanied by the changes that we consider evidences of putrefaction, such as the formation of bacteria and the evolution of fetid gases.

In the treatment of abscesses by the antiseptic method, Mr. Lister believes that he has effected an entire revolution in the course of the disease after the cavity has been opened, and to this I will more particularly allude in my next lecture. But I may here mention that, along with many local advantages, the patient is said to be free from all danger of irritative fever as the immediate consequence, and from hectic at a later stage.

I must state these things explicitly to you, in justice to Mr. Lister, that you may judge fairly of the results of the antiseptic treatment, understanding what it can not do, as well as knowing the advantages claimed for it by its author. It is only just to Mr. Lister, and essential, in order to enable you to form a fair estimate of the results of his method, to remember that he is far from regarding putrefaction as the only cause of suppuration. On the contrary, he has long since pointed out that any antiseptic substance, such as carbolic acid, if applied continuously to the exposed tissues of a wound, stimulates them to granulation, and the granulation to the formation of pus, giving rise to what he has termed "antiseptic suppuration," due to the direct chemical stimulus of the antiseptic. He has also expressed the belief that putrefaction acts in a precisely similar manner in causing suppuration, the products of putrefaction being acrid chemical substances; but that there is this all-important difference between the two cases—that the antiseptic acts only on the part to which it is applied, whereas putrefaction, being a fermentation, extends itself into all the recesses of a wound or abscess, where blood or sloughs, pus or serum, affords a nidus for the development of the bacteria. Further, Mr. Lister has directed attention to the important truth that suppuration, besides being brought about in this manner by the direct stimulus of chemical irritants, may be produced by ordinary inflammation without the access of any external disturbance, putrefactive or otherwise, as in the familiar case of an ordinary deep-seated abscess, the contents of which, when evacuated, are free from putrefaction. This ordinary inflammation he believes to be due to excited nervous action, and the commonest of all causes of it in surgical practice is tension, occasioned by blood or serum being pent up in the cavity of a wound; and he has insisted upon the fact that, in consequence of the irritating influence of the antiseptic material in the spray and sponges, the sanguineo-serous discharge is greater in the earlier periods from a wound treated antiseptically than from one managed in the ordinary way. Hence it is doubly necessary to provide free escape for this serous effusion, which is done by means of drainage tubes; and if these be neglected or inadequate, tension will inevitably result, with corresponding inflammation, and in due time suppuration. Lastly, we must bear in mind that inflammation caused in this manner by tension, like any other ordinary inflammation, will be attended, in proportion to its intensity, by constitutional disturbance or fever.

### WASHINGTON'S TEETH.

The Father of his Country had only one false thing about him. That was a set of false teeth. He wore a double set. I have them in one hand while writing you these lines with the other.

That Washington wore false teeth, and that they were very roughly made, will explain some things in regard to the expression of his contenance, which has been the subject of discussion. There is a marked difference in the mouth as represented in the Peale portait, and in the Stuart portrait, which has given the most general impression to the world of the style of face. In Stuart's portrait, and in all the engravings made from it and repeated by millions, there is a projecting upper lip, giving an expression of *pouting* which Washington himself did not like, because it was not natural to him.

Gen. G. B. McClellan has the original of a medallion head of Washington which the illustrious man preferred so decidedly that he presented it to Mrs. Morris, with the following words in a note:

"The President's compliments accompany the enclosed to Mrs. Morris."

This portrait has been elegantly engraved under the direction of the late Secretary of War. It shows no projection of the upper lip; the mouth is firm and symmetrical, and the appearance of fulness, as in the other, is wanting.

These differences in the expression are easily explained by the fact that Washington lost all his teeth; had a set of artificial teeth made; they were clumsy and irksome, so that it is quite likely they were sometimes worn and often not: that the position of the lips was taken as they appeared at various times; and the whole expression varies with that of the mouth, which is more of an index of the mind than any other portion of the human countenance. Try the experiment, and see for yourself what a difference it makes as you compress or extend the lips. Lavater will give you all the types of man as indicated by the mouth. Other physiognomists have held that there is a direct relation of the muscles of the lips to the upper lobes of the brain, so that, unless repressed or perverted by artificial means, the mouth will naturally intimate some of the most important mental exercises or peculiarities. A man with false teeth, so set as to change the natural shape of his lips, and to prevent their ready play with the varying moods of the mind, will not be fairly handed down to posterity in a picture that gives him a protuberant mouth, unless nature gave him one with his natural teeth.

The history of these false teeth of Washington is not involved in obscurity. And as they are the most nearly personal of anything relating to Washington that survives the grave, they are objects of real interest, and not merely of curiosity. In the Milan Cathedral they preserve with great care, teeth from the head of Abraham, the Father of the Faithful; of Daniel, who stopped the mouths of lions; of John also, and the prophet Elisha; and all these are claimed to be the veritable molars of the men whose bones were buried long ages agone.

But it is not pretended that these are the teeth that Washington had in his head when he had the hatchet in his hand. By the way, if you have any doubt as to the truth of the hatchet story in the early life of G. W., let me just say that I have a piece of the cherry tree which he hacked; of course, the hacking required a hatchet, and thus the historical incident, so valuable in juvenile instruction, is put beyond reasonable dispute. With unreasonable people there is no use of reasoning.

At what period in his eventful life, our great Washington first lost his old teeth and got these new ones, does not appear in any biographies in my possession. But it was not until the year before his death that Washington addressed to Mr. Greenwood, a dentist of New York, the following letter, in which the defects of the work are set forth and improvements suggested with that accurate detail and perspicuity which distinguish the writings of this wonderful man:

PHILADELPHIA, 12th Dec., 1798.

SIR:

Your letter of the 8th came safe, and as I am hurrying in order to leave this city to-morrow, I must be short.

The principal thing you will have to attend to, in the alteration you are about to make, is to let the upper bar fall back from the lower one, whether the teeth are quite straight, or inclining a little in, or a little rounding outwards, this is immaterial; for I find it is the bars alone, both above and below, that give the lips the pouting and swelling appearance—of consequence, if this can be remedied, all will be well.

I send you the old bars, which you returned to me with the new set, because you have desired. But they may be destroyed, or anything else done with them you please, for you will find that I have been obliged to file them away so much above, to remedy the evil I have been complaining of, as to render them useless, perhaps, to receive new teeth. But of this you are better able to judge than I am. If you can fix the teeth (not on the new bars which you have) on the old bars which you will receive with this letter, I should prefer it, because the latter are easy in the mouth—and you will perceive, moreover, that when the edges of the upper and lower teeth are put together, that the upper falls back into the mouth, which they ought to do, or it will have the effect of forcing the lip out just under the nose.

I shall only repeat again that I feel much obliged by your extreme willingness and readiness to accommodate me, and that I am, Sir, Your obed't servant

Go. Washington.

Mr. Jno. Greenwood.

"The letter was written to John Greenwood, of New York, who had a younger brother, William Pitt Greenwood, in Boston, both skilled in the practice of dentistry as then understood.

"The 'bar' referred to took the place of what is now the plate, and was made of ivory carved to an approximate adaptation to the gum, and the teeth were fastened by rivets to the bar. At that time neither the principle of complete support by atmospheric pressure nor by springs was well understood, and the support of an artificial set in the mouth depended somewhat on training of the facial muscles.

"The bulk on the thickness of the bar, together with the action of the muscles in keeping it in place, was apt to give a puff or 'pouting' expression of the face, or some part of it. This, in the case under consideration, was principally evident about the upper lip, as seen in Stuart's portrait of Washington, painted about 1796, while the subject had the set of teeth, the bar of which he had, being at a distance from the dentist, modified so much with his own hands, to obtain comfort, and if possible to correct the objectionable expression mentioned.

"The suggestion in regard to the 'falling back' of the upper teeth, though probably not anatomically correct, even at his age, was evidently made with reference to the same point, as an additional aid in diminishing the pouting expression of the upper lip, and in producing the original symmetry of the face which we find in Houdon's bust, made in 1785, and which is also seen in Peale's portraits.

Jacob L. Williams."

Observe that Washington specifies the pouting expression as the result of these badly fitting teeth. And to look at them, the wonder is that any man ever held them in his mouth five minutes. The teeth are bits of bone, scarcely trying to look like teeth, attached to gold plate, with strips riveted across to strengthen the teeth in place: while coiled wire at the end of the jaws makes a spring, and assists in opening and closing the machine. They are as near to the perfect work of our day in this line, as the old windmill telegraph to a Morse instrument. The Baltimore Dental College has obtained possession of them, and Drs. J. Allen & Son, of this city, preparing a case of their workmanship for exhibition at the Centennial in Philadelphia, have secured the loan of them to put into the foreground of their magnificent display, that the present generation may see what the Father of his Country endured in the times that tried men's souls. He must have been a man of exemplary patience and fortitude, to have borne with these things in his mouth. As to eating with them, or talking with them in, it would seem impossible, but it is probable they answered the purpose very well, for it is not always the handsomest looking machine that does the best work.—*N. Y. Observer.*

